# The Tertiary Hospital Restructuring Project in South Korea: A Reflexive Thematic Analysis of Hospital Directors’ Perspectives on Barriers and Policy Recommendations

**DOI:** 10.3390/healthcare14162469

**Published:** 2026-08-10

**Authors:** Hooyeon Lee, Ji Eun Kim, Kyungin Lee, Dong Jun Kim, Kwangmo Yang, Kyounga Lee, Myung-Il Hahm

**Affiliations:** 1 Department of Preventive Medicine, College of Medicine, The Catholic University of Korea, Seoul 06591, Republic of Korea; 2 Department of Medical Science, Graduate School, Soonchunhyang University, Asan 31538, Republic of Korea; 3 Department of Public Health and Healthcare Management, Graduate School, The Catholic University of Korea, Seoul 06591, Republic of Korea; 4 Health Insurance Research Institute, National Health Insurance Service, Wonju 26464, Republic of Korea; 5 Department of Data Convergence and Future Medicine, Sungkyunkwan University School of Medicine, Suwon 16419, Republic of Korea; 6 College of Nursing, Gachon University, Inchoen 21936, Republic of Korea; 7 Department of Health Administration and Management, College of Medical Science, Soonchunhyang University, Asan 31538, Republic of Korea

**Keywords:** tertiary hospital, health workforce, healthcare delivery system reform, physician assistant, qualitative research, South Korea

## Abstract

**Background**: In September 2024, South Korea launched a nationwide Tertiary Hospital Restructuring Project to refocus tertiary hospitals on severe, emergency, and rare disease care, amid a tertiary care system long burdened by patient concentration and acutely strained by the mass resignation of trainee doctors. In the early phase of implementation, understanding how the decision-makers who translate policy into institutional change perceive the project is essential. **Methods:** We employed a qualitative design using reflexive thematic analysis. Semi-structured, in-depth interviews were conducted with directors from five tertiary hospitals—four in the capital area and one in a non-capital area—selected through purposive sampling. Interviews were conducted between 30 October and 7 November 2025, transcribed verbatim, and analyzed following the six-phase approach of Braun and Clarke. **Results:** Four themes were constructed. First, directors described the project as catalyzing a cultural shift toward a high-severity-centered model, though contested at the departmental level. Second, a severity paradox emerged, in which treating sicker patients worsened profitability while large restructuring costs strained sustainability. Third, referral networks expanded but were constrained by the absence of an integrated information technology (IT) system across the referral network. Fourth, a specialist- and physician assistant (PA)-centered workforce model stabilized care yet remained institutionally unsettled. Participants recommended refined severity criteria, complexity-based reimbursement, a legal framework for PAs, an integrated IT referral system, and network-based training. **Conclusions:** As perceived by the participating directors, the project reshaped organizational culture and clinical case-mix, but its long-term viability depends on financial, infrastructural, and workforce supports that have not kept pace with structural change. Sustaining the reform will likely require a shift from competition toward a collaborative, region-centered network.

## 1. Introduction

While Korea’s National Health Insurance system guarantees universal coverage and access to care, patients retain the legal right to seek treatment at any level of facility—including tertiary hospitals—without a mandatory referral requirement [1]. Despite their designated role in managing severe and rare diseases requiring advanced medical technology, tertiary hospitals continue to be burdened by a considerable volume of mild-condition cases that could be appropriately managed at primary care clinics or secondary hospitals [1,2].

These long-standing structural problems were intensified by the acute workforce crisis of 2024, which exposed the vulnerability of a tertiary care system overly reliant on trainee residents. To protest government healthcare policies—including an increase in the medical school admissions quota—approximately 90% of trainee doctors collectively resigned in early 2024 and remained off duty for a protracted period, only beginning to return in the latter half of 2025 [3,4]. Because trainee doctors constitute a substantial share of the clinical workforce, their abrupt departure created acute shortages, redistributing clinical responsibilities to the remaining specialists and PA nurses [5,6,7]. In the Korean context, a PA nurse is not the separately licensed physician assistant profession recognized in some other countries, but an experienced registered nurse who, in the absence of any distinct physician-assistant license, has by convention taken on duties supporting physicians’ clinical and surgical work. This role is referred to variously as PA, surgeon assistant (SA), or clinical-support nurse in domestic usage, and is rendered in the Korean and international literature as physician assistant nurse (PA nurse) to reflect this country-specific arrangement [8].

To address these challenges and to reduce the system’s heavy reliance on trainee residents that this crisis had exposed, the Korean government launched the comprehensive Tertiary Hospital Restructuring Project in September 2024, aiming to concentrate critical and rare diseases at tertiary hospitals while redistributing low-complexity procedures to lower-tier institutions, including general hospitals and clinics. A central goal of the project is to transition tertiary hospitals away from their heavy reliance on trainee residents toward a specialist-centered care model [9]. As of 2025, all 47 designated tertiary hospitals in South Korea are participating in the restructuring project [10].

While there is broad consensus on the necessity of the project for enhancing healthcare delivery efficiency, significant ambiguity persists regarding its practical implementation and its intended or unintended consequences. Although the policy framework has been clearly articulated, little is known about how it is actually unfolding in practice, and empirical evidence on its early implementation remains scarce. In particular, the perspectives of hospital directors—who make the strategic decisions that determine whether the reform succeeds—have rarely been examined, despite their central role in translating policy into institutional practice.

Consequently, this study aims to qualitatively investigate the perceptions and evaluations of tertiary hospital leadership concerning the current implementation progress, identified barriers, and specific recommendations for policy refinement. Specifically, this study addresses two research questions: (1) How do tertiary hospital directors perceive the early implementation of the restructuring project, including its achievements and the barriers encountered? and (2) What policy refinements do they recommend to support its sustainable implementation? By examining the perspectives of these key decision-makers, this research seeks to provide evidence-based insights to optimize the current framework and ensure the sustainable execution of the restructuring initiative.

Although the project is specific to the Korean context, the challenges it addresses—population aging, the concentration of patients and resources in tertiary hospitals, and an over-reliance on trainee labor—are shared by many health systems. For example, workforce shortages and the concentration of patients in higher-level hospitals have driven comparable reforms elsewhere: England’s NHS Long Term Workforce Plan responds to specialist shortages and an over-reliance on internationally recruited and trainee staff [11], while several OECD countries have introduced payment and governance mechanisms to concentrate complex, high-acuity care in tertiary centres and strengthen referral coordination across care levels [12]. The lessons drawn from Korea’s experience may therefore offer transferable insights for other countries seeking to rebalance their healthcare delivery systems toward higher-acuity, more sustainable tertiary care.

## 2. Materials and Methods

### 2.1. The Tertiary Hospital Restructuring Project

The project aims to concentrate severe, emergency, and rare disease care at tertiary hospitals while redistributing low-complexity care to lower-tier institutions, including general hospitals and clinics. As of 2025, all 47 designated tertiary hospitals in South Korea are participating [9]. The project is organized around five structural domains—clinical care, care coordination, bed management, staffing, and resident training—and participating hospitals are required to submit implementation plans addressing each [13].

In the clinical care domain, hospitals must strengthen their essential specialized functions and raise the proportion of inpatients with conditions classified as appropriate for tertiary care to at least 70%—up from a baseline of approximately 50%. In the care coordination domain, they must establish strengthened back-referral protocols and form formal partnerships with secondary and small- to medium-sized hospitals, supported by additional financial incentives for regional patient transfers. In the bed management domain, hospitals are required to reduce general ward capacity by 5–15%, while expanding the share of intensive care unit (ICU) beds and implementing strategic bed-allocation systems. In the staffing domain, the project promotes a gradual shift away from a trainee-resident-dependent model toward a specialist- and physician assistant (PA)-centered structure. Finally, in the resident training domain, it seeks to improve the quality and density of resident education while reducing reliance on residents’ labor. These structural changes are underpinned by a revised reimbursement framework that raises fees for high-complexity surgery, anesthesia, intensive care, and emergency services.

### 2.2. Study Design and Participants

This study employed a qualitative research design based on reflexive thematic analysis. Of the 47 tertiary hospitals designated nationwide for the 2024–2026 period, hospital directors from five institutions were recruited for in-depth individual interviews—four in the capital area (Seoul and Gyeonggi-do) and one in a non-capital area (Chungcheong-do). Participants were selected through purposive sampling to capture diverse institutional and regional perspectives. Because the participants were the most senior decision-makers of designated tertiary hospitals—an elite, hard-to-access group whose accounts each represent an entire institution—a small number of information-rich cases was considered appropriate for the study aims. The adequacy of the sample was appraised using the concept of information power, which holds that the more relevant information a sample contains for the study aim, the fewer participants are required [14]. The sample offered high information power given the narrow and clearly bounded study aim, the highly specific and knowledgeable participants who occupy a rare strategic vantage point within their institutions, the theoretically informed and reflexive analytic strategy, and the depth and quality of the extended interviews.

Interviews were conducted between 30 October and 7 November 2025, ranging from 60 to 140 min in duration, and were transcribed verbatim. At the time of the interviews, trainee doctors had only just begun to return following their prolonged absence, so the restructuring project was still operating largely under the specialist- and PA-centered arrangements that hospitals had adopted during the workforce shortage. The directors’ accounts therefore reflect this transitional moment, in which the resident workforce was beginning to be reinstated but had not yet been fully reintegrated. This study was approved by the Institutional Review Board (IRB) for Clinical Research (No. SCH 2025-06-005) and conducted in accordance with the Declaration of Helsinki. Written informed consent was obtained from all participants prior to their involvement. The characteristics of the participating hospitals and their directors are summarized in Table 1.

### 2.3. Interview Domains and Questions

The interviews were conducted primarily by the corresponding author, who has experience in qualitative research. The interviewer was professionally acquainted with some of the participants through prior work-related contact but had no personal relationship with any of them; a reflexive stance was maintained throughout to guard against the influence of these professional acquaintances. Participants were identified through purposive sampling and approached directly by the research team, and no invited director declined to participate. All interviews were conducted face-to-face and in Korean, audio-recorded with participants’ consent, and transcribed verbatim. Analysis was carried out on the Korean-language transcripts; only the quotations presented in this article were translated into English by bilingual members of the research team; the translations were then independently back-translated into Korean and cross-checked against the original transcripts by the research team.

Semi-structured, in-depth individual interviews were conducted using an interview guide organized around four domains: (1) the implementation status of the restructuring project, (2) perceived achievements and positive outcomes, (3) barriers and challenges encountered during implementation, and (4) recommendations for policy refinement. The use of open-ended questioning allowed new topics to emerge organically, which were then explored in depth as the conversation developed.

### 2.4. Data Analysis

Transcripts were analyzed using reflexive thematic analysis following the six-phase approach proposed by Braun and Clarke [15,16]. Through a recursive process of data familiarization, code generation, theme development, review, definition, and reporting, the research team interpretively identified patterns of meaning across the dataset. Rather than mapping the findings onto the project’s five administrative domains (clinical care, care coordination, bed management, staffing, and resident training), we developed themes inductively from the data, generating four overarching themes that recurred across domains. To enhance the trustworthiness and interpretive depth of the findings, the researchers maintained a reflexive stance throughout, and emerging interpretations were discussed and refined collaboratively through regular team meetings.

Coding proceeded inductively from the transcripts: we first generated initial codes close to participants’ own language, then grouped related codes into candidate themes through iterative comparison across the dataset, and refined these into four overarching themes that were reviewed against the coded extracts and the full transcripts. Although the results are presented under the headings of achievements, barriers, and recommendations for readability, these headings serve as an organizing device rather than as the themes themselves; the four themes are interpretive constructs that cut across the project’s five administrative domains and the interview topics, capturing tensions—such as the severity paradox—that recurred regardless of the domain under discussion. Divergent and contradictory accounts were actively sought during analysis and retained in the final themes rather than smoothed over. In line with the reflexive orientation of the analysis, we also considered our own positionality. The research team comprised academic researchers with backgrounds in health services research, health policy, and nursing, and had no affiliation with or managerial relationship to the participating hospitals; the team therefore approached the study as external investigators rather than as stakeholders in hospital operations. The interviews, coding, and theme development were conducted collaboratively by team members specializing in health services research, health policy, and nursing. We recognize that these disciplinary orientations shaped our analytic lens: our training in health services research and health policy directed particular attention to system- and policy-level dynamics—such as reimbursement, referral coordination, and workforce structure—while our nursing perspective heightened our sensitivity to workforce-related themes, including the evolving role of PA nurses. To mitigate the influence of these preconceptions, interpretations were continually questioned and refined collaboratively throughout the analytic process.

## 3. Results

Four overarching themes were constructed through the thematic analysis: (1) a catalyzed but contested cultural shift; (2) the gap between reimbursement and financial sustainability; (3) coordination pursued without adequate infrastructure; and (4) a workforce and training model caught in transition. These themes cut across the five domains of the restructuring project, reflecting the tensions that recurred most consistently across participants.

### 3.1. Redefining the Tertiary Hospital: Institutional Consensus, Departmental Contestation

#### 3.1.1. Achievements

Participants consistently described how the project catalyzed a shift in internal awareness and organizational culture toward a high-severity-centered model. All five participants noted that the broader workforce crisis arising from the trainee doctors’ mass resignation had, paradoxically, created room to realign their institutions around their intended function, and that the reorientation toward severe, high-complexity, and rare diseases came to be understood as both the proper role of a tertiary hospital and a financially defensible direction. This recognition provided a basis for directing human and material investment toward essential specialties.

*“The crisis paradoxically opened up room for us to adjust our patient mix and reorganize our care system around high-severity cases.”*

(P1)

*“It became a turning point for our entire faculty and staff to recognize that quality of care matters more than sheer volume.”*

(P3)

#### 3.1.2. Barriers and Recommendations

This consensus, however, was contested at the level of individual departments. Participants reported intensifying avoidance of high-liability, low-reimbursement specialties such as obstetrics and neurosurgery, alongside growing dissatisfaction among departments that treat a relatively high proportion of patients with conditions not classified as tertiary-appropriate under the restructuring project—such as ophthalmology, otolaryngology, orthopedics, dermatology, and rehabilitation medicine—which generated internal conflict and demotivation. Several directors also feared that the project risked narrowing the tertiary general hospital’s identity away from its comprehensive mission of care, education and training, and research, toward a near-exclusive focus on clinical care. Participants recommended department-specific severity indicators that account for complexity, difficulty, and skill rather than excluding lower-severity departments outright, together with recognition of teaching and research contributions and national public-awareness efforts to curb the inflow of mild cases.

*“Departments such as OB-GYN and neurosurgery, where legal liability is exceptionally high but compensation remains low, are being avoided more and more—while departments characterized by lower severity—including ophthalmology, otolaryngology, orthopedic surgery, dermatology, physical medicine and rehabilitation, and endocrinology and metabolism grow increasingly dissatisfied, creating internal conflict.”*

(P3)

*“It feels as though we have shifted away from our combined mission of education, research, and care toward being centered almost entirely on clinical care.”*

(P1)

### 3.2. The Severity Paradox: Treating Sicker Patients, Earning Less

#### 3.2.1. Achievements

Across participating hospitals, the proportion of conditions classified as appropriate for tertiary care reportedly increased by 2.5% to 17% following the project, as reported by the directors based on their institutions’ internal data rather than independently verified by the research team, although participants cautioned that this partly reflected a decline in mild cases rather than an absolute rise in severe cases. Government subsidies—including policy add-ons for inpatient fees, add-on payments for high-severity surgery, and 24 h care support funds—were widely credited with offsetting declining revenue, and the fixed-amount bed-loss compensation provided substantial financial support for hospital operations.

*“The bed-loss compensation, provided as a fixed amount, was a great help in sustaining our hospital’s operations.”*

(P4)

#### 3.2.2. Barriers and Recommendations

Nonetheless, financial sustainability emerged as a particularly pervasive challenge. This financial strain was reported across both private and public institutions, indicating that it was not confined to a particular ownership model but reflected structural features of the reform itself. Participants observed that treating more high-severity patients paradoxically worsened profitability, while the high upfront cost of restructuring—approximately 10 billion KRW (approximately USD 6.5 million, at KRW 1530/USD as of July 2026) per institution for demolition, facility expansion, equipment, and staffing (as reported by the directors based on their institutions’ internal data rather than independently verified by the research team)—strained liquidity, especially as bed occupancy fell during construction. In addition, the decline in outpatient visits for mild conditions, although an intended effect of the policy, further eroded revenue and compounded the financial strain. They further criticized the current fee schedule for inadequately reflecting clinical complexity, the inconsistency of ICU-ratio standards across evaluation schemes, and uniform bed-ratio requirements ill-suited to cancer-centered hospitals with inherently low ICU demand. Recommendations included a larger add-on differential for high-severity conditions, a stable long-term financial support framework, and support that accounts for region-specific conditions, including medically underserved areas.

*“As we treat more high-severity patients, our profitability actually worsens. The add-on differential for high-severity conditions needs to be substantially larger.”*

(P2)

*“Support for initial investment costs is insufficient. Just converting general beds into ICU beds—along with expanding facilities, equipment, and staffing—cost around 10 billion KRW.”*

(P4)

*“A uniform bed-ratio standard simply isn’t realistic for a cancer-centered hospital, where ICU demand is inherently low.”*

(P5)

### 3.3. Coordination Without Shared Infrastructure

#### 3.3.1. Achievements

Participants reported expanding the functions of their Care Coordination Centers, increasing staffing to build specialized referral and back-referral systems, and strengthening regionally complete partner-hospital networks through site visits, education, and consulting. Back-referral processes for relatively stable patients became established, and several institutions introduced fast-track pathways—some operating dedicated referral slots distinguishing initial referrals from re-referrals—to prioritize partner-hospital patients.

*“We run our fast-track in two streams—initial referrals and re-referrals after a back-referral—so that partner-hospital patients can be prioritized.”*

(P4)

#### 3.3.2. Barriers and Recommendations

The central barrier participants identified was insufficient infrastructure and staffing across the referral network. They emphasized that genuine continuity of care can be achieved only when both tertiary hospitals and their partner hospitals have the necessary infrastructure and dedicated personnel in place. Participants also noted that partner hospitals often functioned as mere extensions of tertiary hospitals rather than as active care partners, and that patients tended to misunderstand the back-referral system, perceiving referral back as being turned away during treatment. Accordingly, they recommended providing legal and financial support to enable partner hospitals to actively accept referred-back patients and implementing public education and outreach on the cooperative care system to improve patient understanding.

*“Continuity of care can only be fully guaranteed when both the tertiary hospital and its partner hospitals have the necessary infrastructure and dedicated staff in place.”*

(P3)

*“Partner hospitals also need legal and financial support so that they can actively accept patients referred back from tertiary hospitals, rather than serving as mere extensions of them.”*

(P1)

*“Patients often don’t understand what tertiary hospitals are for or how back-referral works—they feel they’re being turned away in the middle of treatment. We need nationwide public education about the cooperative care system.”*

(P3)

### 3.4. A Workforce and Training Model in Transition

#### 3.4.1. Achievements

Participants reported the stabilization of a care delivery model centered on specialists and PA nurses and lessened reliance on residents. PA teams were organized with systematic role division, performing core clinical-support functions in operating rooms, emergency departments, and essential specialties.

*“Our PA teams now handle core clinical-support tasks in the operating room and emergency department, which has eased the burden on our specialists considerably.”*

(P5)

#### 3.4.2. Barriers and Recommendations

Yet this model remained institutionally unsettled. Participants noted that securing and retaining a sufficient number of specialists remained extremely challenging, raising concerns about the long-term viability of a specialist-centered model. This difficulty was particularly acute outside the capital area, where participants reported that the concentration of personnel in the capital region exacerbated burnout and attrition among their existing specialists.

*“In terms of staffing, the collaboration between specialists and PAs helped minimize gaps in patient care, but securing specialists remains difficult in reality.”*

(P1)

*“Personnel are concentrating in the capital region, leading to burnout and attrition among existing specialists.”*

(P5)

During the workforce crisis, PA nurses had taken on substantially expanded clinical-support roles as a matter of practice, even though no detailed legal framework yet defined their scope of practice or qualifications; this de facto expansion persisted throughout the interview period. At the same time, the ambiguous role and legal standing of PA nurses—compounded by overlapping duties following residents’ return—heightened employment insecurity and produced costly personnel redundancy.

*“With residents now returning, PA roles overlap with theirs and are being scaled back, so employment insecurity among our PAs is growing.”*

(P1)

A more fundamental tension also emerged between the project’s high-severity orientation and residents’ need for broad clinical exposure, raising concern that tertiary hospitals concentrating on severe cases could no longer provide well-rounded training.

*“Under the restructuring project, tertiary hospitals are no longer a suitable environment for resident education.”*

(P3)

*“It would be far more efficient for residents to first encounter many moderately complex cases at secondary hospitals, then take on highly complex training at tertiary general hospitals.”*

(P2)

To address these challenges, participants offered recommendations targeting each group of the clinical workforce. For PAs, they recommended clearly defining their scope of practice, establishing a formal legal framework and a nationally standardized education system, and reforming reimbursement to reflect their contributions [17]. For specialists, they emphasized improving working conditions to ease the heavy workload that hampers their recruitment and retention. For trainee doctors, they recommended introducing competency-based evaluation of training and developing network-based training programs that span secondary and tertiary institutions, so that those concentrated in high-severity tertiary settings could still gain broad clinical exposure.

## 4. Discussion

This study examined how tertiary hospital directors perceived the early implementation of Korea’s tertiary hospital restructuring project. Across four themes, a consistent pattern emerged: the directors perceived the project as having reshaped organizational culture and clinical case-mix, but its long-term viability is constrained by financial, infrastructural, and workforce conditions that have not yet caught up with the pace of structural change.

The first theme indicates that cultural buy-in, though substantial, is unevenly distributed across departments. While directors and senior staff broadly embraced the high-severity orientation as the proper role of a tertiary hospital, departments treating a higher share of lower-severity patients experienced demotivation and internal conflict, and several directors feared an erosion of the hospital’s comprehensive mission of care, education, and research. Sustaining cultural buy-in will therefore require severity metrics sensitive to departmental difference and explicit safeguards for the educational and research missions that participants feared losing.

The second theme positions financial sustainability as the central fault line of the reform. Participants described a severity paradox in which treating more severe patients eroded profitability, while large upfront investments and a policy-induced decline in outpatient revenue compounded the strain. As long as these structural disincentives persist, even willing institutions face financial pressure that undermines the reform. A complexity-based reimbursement system and a stable medium- to long-term financing framework—together with support tailored to medically underserved regions—therefore appear essential rather than optional. This severity paradox also resonates with international experience of payment reform. Across many high-income systems, health care has been moving from volume-based payment toward value- and complexity-oriented models in the search for a financially sustainable system, and the success of such reforms depends on how well reimbursement reflects patients’ clinical complexity and the actual cost of the care delivered [12,18].

The third theme shows that referral networks cannot mature into genuine care coordination without an integrated IT system across the referral network and accountable, adequately resourced partner hospitals. Participants emphasized that continuity of care depends on both the tertiary hospital and its partner hospitals having the necessary infrastructure and dedicated staff, and that partner hospitals require legal and financial support to actively accept back-referred patients rather than serving as mere extensions of the tertiary hospital. Coordination mandated without this infrastructure risks remaining a quantitative metric rather than a qualitative reality.

The fourth theme reveals a workforce model in transition, in which the legal vacuum surrounding PAs and the unresolved tension between specialization and resident training generate instability that requires legislative and educational action led by the state rather than individual institutions. Participants further highlighted that securing and retaining specialists remained difficult—particularly outside the capital area, where the concentration of personnel in the capital region exacerbated burnout and attrition—and that tertiary hospitals concentrating on severe cases may no longer provide the broad clinical exposure residents need.

Taken together, the four themes point to a common problem of sequencing: within this voluntary programme, the operational requirements that participating hospitals undertook—raising the severity mix, reducing general beds, expanding referral networks, and shifting to a specialist- and PA-centered model—moved ahead of the legal, financial, and infrastructural frameworks needed to sustain them. This sequencing gap, rather than any single barrier, emerges as the central threat to the reform, and aligning the pace of these requirements with that of enabling supports therefore appears to be a precondition for its success.

These findings carry particular significance because the restructuring project was implemented amid an acute absence of trainee doctors following the 2024 mass resignation [3,4]. What might otherwise have been a gradual, voluntary transition away from a resident-dependent model became, in practice, an abrupt necessity, as hospitals were compelled to sustain clinical operations with specialists and PA nurses in the near-total absence of residents. In this sense, the directors’ accounts capture not merely the planned effects of a policy but the lived experience of institutions adapting to a workforce shock and a structural reform simultaneously. This dual context lends the specialist- and PA-centered model both greater urgency and greater fragility: the model emerged as an immediate coping mechanism, yet its long-term sustainability remains contingent on the very legal, educational, and financial supports that participants identified as still lacking. A large-scale return of trainee doctors could create renewed instability through role overlap and personnel redundancy, suggesting that the crisis-driven workforce reconfiguration cannot be sustained without deliberate national-level institutionalization. Even though trainee doctors began returning in the latter half of 2025, this problem has remained unresolved: with the legal scope of PA practice still being finalized, hospitals continue to face considerable confusion over how to redistribute duties between returning trainees and the PA nurses who had filled their roles [17]. Since the interviews were conducted, this legal gap has begun to be addressed: building on the Nursing Act enacted in 2024, the Ministry of Health and Welfare promulgated the Rules on the Performance of Physician-Support Duties by Nurses and the accompanying Notification in July 2026, which for the first time specify the eligible institutions and nurses, qualification requirements, and permitted scope of practice for physician-support nursing [19].

This study has several limitations. The findings rely on the perspectives of institutional leaders and capture a single point in time in a rapidly evolving policy environment; while the participants’ strategic position as institutional decision-makers offers a distinctive vantage point, their accounts may not reflect the experiences of frontline clinicians, PA nurses, residents, patients, or partner hospitals. In addition, the study drew on only five institutions—although these were purposively selected to maximize information power rather than statistical representativeness, with each director providing an information-rich account of an entire institution [14]. Future research incorporating these perspectives, and tracking changes over time, would provide a more complete account.

## 5. Conclusions

As perceived by the participating directors, the restructuring project reshaped organizational culture and clinical case-mix at Korean tertiary hospitals, but its long-term viability depends on financial, infrastructural, and workforce supports that have not kept pace with structural change. Sustaining the reform is likely to require a shift from a competition-based system toward a collaborative, region-centered network, underpinned by a complexity-based reimbursement and stable long-term financing framework, a formal legal framework and standardized education system for PAs, an integrated national IT referral system, and sustained public engagement to build trust in the tiered delivery system. Among these, reforming the reimbursement framework to reward clinical complexity stands out as a particularly important priority, as the other supports may not be sufficient to sustain the reform unless the severity paradox is addressed so that treating sicker patients is no longer financially penalized.

## Figures and Tables

**Table 1:** Characteristics of participating tertiary hospitals and their directors (N = 5).

Characteristic	P1	P2	P3	P4	P5
Region	The capital area (Seoul)	The capital area (Seoul)	The capital area (Seoul)	The capital area (Gyeonggi-do)	The non-capital area (Chungcheong-do)
Position	hospital director/president	hospital director/president	hospital director/president	hospital director/president	hospital director/ president
Interview duration (min)	102	107	138	65	83
Bed size	≥1500	1000–1499	<1000	<1000	1000–1499
Ownership	private	private	private	private	public
Tenure in leadership (years)	≥5	<5	<5	≥5	<5

Note: To protect participant confidentiality given the small sample and the public visibility of tertiary hospital directors, institutional and personal characteristics are reported only in aggregated, non-identifying categories. Region: capital area/non-capital area. Bed size: ≥1500/1000–1499/<1000 beds. Ownership: public/private. Tenure in leadership is reported in broad categories (≥5/<5 years), where tenure denotes cumulative years in director-level and equivalent senior administrative roles, including prior positions such as chief of planning and coordination or deputy director for clinical affairs.

## Data Availability

The qualitative data generated and analyzed during the current study are not publicly available to protect participant confidentiality but are available from the corresponding author on reasonable request.
